# 

**Published:** 1910-07-02

**Authors:** 


### Boric Acid Poultice.

Mix a tablespoonful of cold-water starch and a teaspoonful of boric acid with a little water; add the mixture to a pint of boiling water and stir the whole until a uniform mucilaginous mass is formed. When cold, spread the jelly thickly on cotton and cover it with a piece of muslin. Then apply to the part. A good plan is to put on the poultice at bedtime and to remove it in the morning. It is useful in acute and subacute skin affections to cleanse and soothe prior to the application of ointments.

### Retention of Urine.

If acute urethritis be the cause, catheterisation must be avoided on account of the danger of extension of the infection and the intense pain. Morphia and the hot bath are usually successful; if not, supra-pubic puncture must be done. In cases of stricture the hot bath must be given a fair trial, and then a large-sized silver catheter. Never use small silver instruments; the smallest soft catheters may be tried, generally unsuccessfully. Next try the Gouley catheter with the whalebone guides, several of which should be passed as far as the face of the stricture. With patience and careful manipulation one of them may enter and a silver catheter passed along the guide. If unsuccessful, it is best to do a supra-pubic puncture, and later to try catheterisation again, possibly with an anæsthetic, before resorting to any cutting operation.—*Mr. Percy Sargent.*

### COMING EVENTS OF THE WEEK.

Saturday, July 2.—League of Mercy. Lambeth, Kennington District: Mrs. Hornby Steer, At Home, 52 Avenue Road, Regent's Park, N.W., 4 p.m.
Dartford District: Mrs. Carl Jay, At Home, Blendon Hall, Bexley, 3.30 p.m.
Opening Harrogate Baths Extension, Harrogate.

Sunday, July 3.—Visiting day at the Hospitals.

Wednesday, July 6.—Ealing Hospital, Matlock Lane, Laying of Foundation Stone by Lord Rothschild, 6 p.m.

Thursday, July 7.—Royal College of Surgeons. Election of Members to Council (four vacancies), 3 p.m.
Guy's Hospital Medical School, Garden Party, 3.15.
League of Mercy, Camberwell North District: Mr. S. Hoffnung-Goldsmid, At Home, 35 Chesham Place, 7 p.m.

### GIFTS AND BEQUESTS.

MRS. CLARA SEYMOUR, of Nottingham, has bequeathed £100 to the Nottingham Hospital for Women.

THE Fishmongers' Company has sent 25 guineas to the British Lying-in Hospital, Endell Street, W.C.

MR. GEORGE AARON ISAACS, of Mile End, has left £100 for division between ten London hospitals and charities.

THE Royal Dental Hospital, Leicester Square, has received £250, duty free, under the will of Miss Emma Brandreth.

MR. WILLIAM JONES, of Poplar, has bequeathed £200 each to the City of London Hospital for Diseases of the Chest, the Surgical Aid Society, and to the West Ham Hospital.

THE REV. EDWARD FORBES, of Clifton, founder and chaplain of the Clevedon Convalescent Home from 1881, left £25 to the Clevedon Convalescent Home.

MRS. MARIA SHAW, of Standon, Staffordshire, has bequeathed £200 each to the Stafford Infirmary and the North Stafford Infirmary at Hartshill, Stoke-on-Trent.

THE late Sir Walter Scott, of Newcastle-on-Tyne, has left £500 to the Fleming Memorial Hospital for Sick Children, Newcastle; £1,000 to the Royal Victoria Infirmary, Newcastle.

MISS ELLEN ELIZABETH BOND, of Lancaster, has left a sum of £10,000 with trustees to build a sanatorium near Lancaster for the relief and cure of consumptives in Lancaster. It will be called the James Bond Sanatorium for Consumptives.

THE Treasurer of St. Bartholomew's Hospital acknowledges the following further contributions towards the special fund—namely, the Worshipful Company of Merchant Taylors (second moiety of grant of £1,000), £500; the Worshipful Company of Cutlers, £52 10s.; Sir John Ellerman, £52 10s.; Waterlow and Sons, Limited, £52 10s.

MISS ELIZABETH CHAMBERS, of St. Andrew's Square, Hastings, who died on May 23, aged ninety, left estate of the gross value of £36,731, with net personalty £36,174. The testatrix bequeathed £10,000 to King Edward's Hospital Fund; £5,000 to Guy's Hospital, London; £1,000 each to the Royal Free Hospital, Gray's Inn Road; the Cancer Hospital, Brompton; the London Hospital, Whitechapel Road; the Hastings, St. Leonards, and East Sussex Hospital; and the Buchanan Hospital, St. Leonards-on-Sea; and £500 to the Hastings and St. Leonards Homœopathic Dispensary. The residue of her estate she left to Mr. William Freeman Beresford absolutely, but in the event of his predecease to Guy's Hospital, London.

H.S.H. PRINCE FRANCIS OF TECK has received £1,000 from Mr. Edmund Davis, £525 from "Anonymous," £100 from Messrs. Carrington & Co., and £100 from "R. D. W.," in response to his appeal on behalf of the Middlesex Hospital.

### APPOINTMENTS.

H. NEVILLE CROWE, B.Ch., M.B., M.R.C.S., L.R.C.P., has been appointed honorary anæsthetist, and R. T. Slinger, M.B., Ch.B., F.R.C.S., has been appointed honorary pathologist, to the Worcester Infirmary.

MR. FRANKLIN EMRYS-JONES, B.A., L.M.S.S.A., of Newhall Street, Birmingham, has been appointed Honorary Radiographer to the West Bromwich District Hospital. Mr. Emrys-Jones already holds similar appointments at the General Hospital, Birmingham, and the Guest Hospital, Dudley.

### JOTTINGS.

THE following letter has been received by Prince Francis of Teck in connection with the special appeal on behalf of the Middlesex Hospital :—

A LETTER FROM THE QUEEN.

"Marlborough House, Pall Mall, S.W.,
"June 25, 1910.

"SIR,—The Queen commands me to forward a cheque for one hundred pounds as a donation to the fund which your Serene Highness is raising to meet the pressing needs of the Middlesex Hospital. And I am further directed to add the expression of her Majesty's best wishes for the success of your endeavour on behalf of an institution which has done very much to alleviate the sufferings of so many in urgent need of assistance.

"I beg to remain, Sir,
"Your obedient servant,
"A. NELSON HOOD,
"Treasurer to her Majesty."

THE total amount in donations to the Hospital Sunday Fund received to date is £31,000.

H.M. THE QUEEN has been graciously pleased to become patron of the Chelsea Hospital for Women.

THE King has been pleased to incorporate under a royal charter the Cancer Hospital (Free), Fulham Road, S.W.

THE QUEEN has given permission that the new maternity ward at St. Thomas's Hospital should be named "Mary" after her Majesty.

THE wards, casualty department, and operating theatre at the West Bromwich District Hospital have been entirely furnished with aseptic furniture, which has been purchased and paid for with the £500 generously given for that purpose by its President (Mr. F. Dudley Docker, J.P.).

BY kind permission of the Lord Mayor and the Lady Mayoress, the annual meeting of the East End Mothers' Lying-in Home (No. 394-98 Commercial Road, London, E.), postponed from May 11 until after the late King's funeral, will now be held on Monday, July 11, at the Mansion House, at three o'clock.

AT a meeting of Jews held last week at the Pavilion Theatre, Mile End Road, Dr. A. Gaster presiding, it was resolved to proceed with a scheme to establish a hospital for Jews in the East End as a memorial to the late King, and to petition the King to allow it to be called "The King Edward Jewish Memorial." The chairman stated that a piece of land had already been purchased and paid for.

THE laying of the foundation stone of the new Ealing Hospital was fixed for Wednesday, July 6, by H.R.H. the Princess Christian of Schleswig-Holstein, who had graciously promised to perform the ceremony. This engagement has, of course, been cancelled in consequence of the lamented death of his late Majesty. The Committee, however, has decided that to postpone the laying of the stone would cause considerable inconvenience, and therefore the arrangements will be carried through as far as possible as originally intended. The Committee has obtained a promise from Lord Rothschild to lay the stone, his lordship taking the place of the Princess.

### TO HOSPITAL SECRETARIES AND OTHERS.

**WE invite contributions from any of our readers, and shall be glad to pay, according to length, for items of news for the institutional section (including appointments, resignations, gifts, etc.) which we may publish. All payments for manuscripts used are made as early as possible after the beginning of each quarter.**

